# Metacognition in Japanese macaques (*Macaca fuscata*): does impulsivity explain unnecessary looks in the tubes task?

**DOI:** 10.1007/s10071-024-01879-1

**Published:** 2024-05-28

**Authors:** Lorraine Subias, Noriko Katsu, Kazunori Yamada

**Affiliations:** Graduate School of Human Sciences, Osaka University, Suita, Japan

**Keywords:** Metacognition, Impulsivity, Passport effect, Japanese macaques, Tubes task

## Abstract

Potential metacognitive abilities, such as monitoring and controlling cognitive processes, have been revealed in some primate species. In the tubes task, apes and macaques showed higher content-checking behavior when unaware of a reward’s location, but they also periodically inspected the tubes when aware, especially when a more appealing reward was involved. Some attribute this to the pleasure of looking at the reward. This study investigates whether the unnecessary tube-checking behavior observed in nine wild Japanese macaques, previously tested for metacognition using the tubes task, can be solely attributed to impulsivity. The macaques’ propensity to look inside a single tube containing food they cannot immediately reach was measured and compared to their behavior in the tubes task. Results indicated that looking inside the baited tube increased as reward quality improved. However, macaques displaying unnecessary tube inspections in metacognitive tests showed less impulsivity to look. This intriguing result counters the notion that excessive looking in the tubes task is solely due to impulsive looking, prompting us to advocate for further research into the relationship between inhibition and metacognitive performance.

**Supplementary Information:**

The online version contains supplementary material available at 10.1007/s10071-024-01879-1.

## Introduction

Metacognition allows individuals to be aware of what they know. Investigating metacognition in animals can uncover consciousness mechanisms and origins. The tubes task, introduced by Call and Carpenter (2001), tests metacognition through information-seeking behavior: a food reward is hidden in one of several tubes, and subjects either know or do not know its location. Apes and macaques exhibit a metacognitive-like response by looking inside the tubes significantly more in the “unknown” compared to “known” condition (Call 2010; Gazes et al. 2023; Hampton et al. 2004; Marsh and MacDonald 2012; Marsh 2014; Rosati and Santos 2016; Subias et al. 2024). Interestingly, apes and rhesus macaques still looked in the “known” condition from time to time.

In a previous experiment (Subias et al. 2024), we tested free-ranging Japanese macaques on the tubes task, using a protocol similar to Call (2010). Macaques looked inside the tubes more when they did not know the reward location but still looked in the “known” condition in 44% of trials. Additionally, some increased looking when a more appealing reward was involved and decreased looking when the effort required was increased.

It is unclear why monkeys and apes engage in unnecessary looking, and why unnecessary looking would increase with the reward quality and decrease with the cost of looking. Call’s proposed explanation, the “passport effect,” suggests subjects want to confirm their memory, especially when stakes are high and the effort to do so is minimal, implying that checking is driven by a metacognitive process. Alternatively, monkeys might solve the tubes task through non-metacognitive mechanisms, such as associative learning or response competition, with unnecessary looking arising from a desire or impulse to view the reward. Perner (2012) suggested looking at a reward to be inherently pleasurable, and using a highly preferred reward would make it more challenging to resist looking. Macaques have undergone testing in various inhibitory control tasks (Loyant et al. 2022; MacLean et al. 2014). Notably, the emotional Stroop effect has been demonstrated in Japanese macaques, revealing impaired accuracy when photographs of preferred food were used as stimuli (Hopper et al. 2021). Yet, despite its relevance, Perner’s suggestion remains unexplored.

The present study tests whether monkeys’ looking in the “known” condition of the tubes task could be attributed to a desire to look at the reward. Nine Japanese macaques previously tested on the tubes task (see Subias et al. 2024 for details) were tested for impulsivity in looking inside a single tube containing food, using low- and high-quality rewards. Contrary to the tubes task, we used one tube and made monkeys wait ten seconds before retrieving the reward, eliminating any competition between monkeys’ drive to select the tube and their inclination to look inside. This approach aimed to prevent the urge to select from overshadowing any potential drive to look, to better assess if such a drive exists. Given the single-tube setup, monkeys cannot make mistakes; thus, there is no need to look. However, if subjects have a drive to look at the reward, they should look no matter the number of tubes, and even more so with high-quality rewards. Additionally, if this impulse drove unnecessary looks in the tubes task, we expected individuals who looked most often in the “known” condition of the tubes task to also look most in the one-tube situation. Therefore, we should observe a positive correlation between monkeys’ looking frequencies in the impulsivity test and the tubes task.

## Method

### Subjects

Tests were conducted on nine Japanese macaques from a free-ranging group in Awajishima, Japan (34°14′41.5"N, 134°52′59.6"E), provisioned by the Awajishima Monkey Center since 1967. The group, estimated at 126 members, received wheat and soybeans thrice daily.

Subjects included eight adult males and one female (> seven years old; Online Resource 1), previously tested for metacognition using the tubes task (Subias et al. 2024). Impulsivity tests were conducted from June to July 2023, 9:30 a.m. to 5:00 p.m., in the feeding area where macaques ranged freely. Subjects freely approached and manipulated the apparatus.

### Testing location and apparatus

A four-tube and one-tube apparatus were used at two testing locations established to test metacognition (Subias et al. 2024). Three subjects were tested at location 1, one at location 2, and the remaining five at both locations opportunistically (Online Resource 1). At both locations, the experimenter (E) stood inside a hut with the apparatus, and the subject interacted through a wire mesh, situated on a wooden platform 103 cm above the ground at location 1, or directly on the ground at location 2.

The apparatus of the two tasks was the same; only the number and position of tubes changed. Both apparatuses included a wooden board (600 mm × 400 mm), with one or four PVC tubes and a transparent PVC screen (550 mm × 320 mm). Tubes (45 mm diameter, 200 mm length) were affixed to the wooden board, with the four-tube apparatus tubes placed approximately 55 mm apart, parallel to each other. In the one-tube apparatus, the tube was centered on the board. A transparent screen, when lowered, prevented physical contact with the tube while allowing visual inspection. Monkeys could grab and tip the tube over to make its content fall. White PVC boxes (55 mm × 85 mm × 43 mm) were placed beneath the board to raise the apparatus to different levels, ranging from level 1 (direct placement on the table, no boxes used) to level 6 (215 mm high), with each level elevation being 43 mm.

### Titration procedure

The tubes task protocol used to test metacognition (Subias et al. 2024) was reused to determine a height at which subjects would be willing to look inside tubes even when unnecessary. For metacognition, monkeys were tested at low- and high-cost levels (i.e., apparatus height was manipulated) to assess the cost effect on looking behavior. The low-cost level prompted frequent tube inspection (50%–75% of trials), while the high-cost level reduced looks (25%–50% of trials). Given the infrequent occurrence of unnecessary looks at the high-cost level, we tested impulsivity to look at the low-cost level. Each session began with a titration procedure to determine the appropriate low-cost level. Using a four-tube apparatus, we set the level most often used for low-cost conditions in previous metacognitive tests (Subias et al. 2024). Each subject received eight trials, divided equally between known and unknown conditions, using low- and high-quality rewards (same as for impulsivity test). In the *known* condition, after presenting the reward, E inserted her finger into the baited tube, clearly indicating the tube with the reward. In the *unknown* condition, after presenting the reward, E passed it between hands to conceal its location. She then inserted fingers into all tubes, discreetly leaving the reward in one. If a) the monkey looked in less than 50% of known trials, the apparatus was raised; if b) the monkey looked in every trial, the apparatus was lowered. This process continued until the monkey looked between 50%–75% of known trials.

### Impulsivity-to-look test

Subjects were already familiar with the procedure and knew how to obtain food from tubes: no training was required. The one-tube apparatus was placed against the hut’s wire mesh (tube perpendicular to the subject) behind a transparent screen. The screen prevented subjects from reaching the tube while allowing them to look inside it. E presented the reward for 3 s, placed it inside the tube, and, after a 10 s delay, removed the screen, allowing the subject to retrieve the reward. A new trial began immediately thereafter.

We tested two conditions:

*Low-quality reward*: Monkeys’ less preferred food was used: a piece of sweet potato or carrot.

*High-quality reward*: Monkeys’ most preferred food was used: a peanut.

Monkeys’ preferences were assessed using food preference tests.

Each subject underwent 12 consecutive trials in one go under each condition. Four subjects (randomly chosen) were tested with low-quality reward first and high-quality reward second, while the other five experienced the opposite order (Online Resource 1). After testing a subject in one condition, a minimum one-week interval preceded testing the same subject in the second condition to minimize the impact of learning in the second session.

During each trial’s 10 s waiting time, we assessed looking behavior by scoring a “look” when subjects lowered their heads to align their eyes with the tube opening. Looking proportions were calculated by dividing the number of trials with looks by the total trials. E scored behaviors in real-time and verified them through video recordings.

### Data analysis

A paired t-test compared looking proportions between low- and high-quality conditions in the impulsivity test, accompanied by the calculation of the effect size (Cohen’s d). Given non-normal distribution and a small sample size, Spearman’s correlation test examined the relationship between monkeys’ looking proportions in the impulsivity test and the tubes task. Cocor package for R (Diedenhofen and Musch 2015) was used to compare the correlation coefficients. We used Dunn and Clark’s z, designed for the comparison of overlapping and dependent correlations, to make the comparison. Yubisashi, who did not differentiate between known and unknown conditions in the tubes task (Subias et al. 2024), was excluded from the correlation analysis but included in other analyses.

To ensure reliability, a second observer scored 20% of the analyzed trials from video recordings. Inter-observer reliability was excellent (Cohen’s kappa = 0.8). All analyses were conducted using RStudio 4.1.0.

## Results

**Fig. 1:**
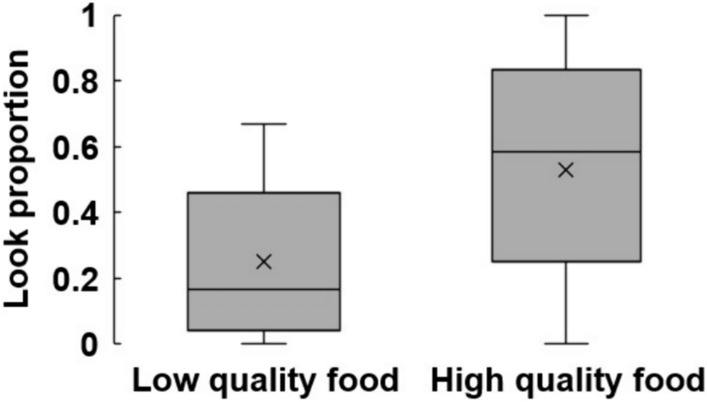
Comparison of looking proportion between low- and high-quality reward. x represents the average value, the middle line represents the median, the upper and lower end of the box represent respectively the third and first quartiles, and the end of the whiskers shows the maximum and minimum values

All nine monkeys made at least one look in at least one condition during the 10 s waiting time, confirming that monkeys have some drive to look at the reward (Table 1). On average, monkeys looked significantly more often when using a high- than low-quality reward (Fig. 1, *t* (8) = − 2.31, *p* = 0.049), with a large effect size (*d* = − 0.941).

**Table 1:** Look proportions in Tubes task and Impulsivity test

	Low-quality reward		High-quality reward	
	Tubes task	Impulsivity test	Tubes task	Impulsivity test
Gaara	0.57 (12/21)	0.17 (2/12) level2	0.40 (8/20)	1.00 (12/12) level2
Gattsu	0.57 (21/37)	0.08 (1/12) level2	0.50 (16/32)	0.17 (2/12) level3
Kikuhime	0.60 (12/20)	0.08 (1/12) level3	0.55 (11/20)	0.00 (0/12) level3
Manta	0.44 (7/16)	0.33 (4/12) level2	0.40 (6/15)	0.92 (11/12) level3
Paku	0.17 (4/23)	0.67 (8/12) level4	0.33 (9/27)	0.42 (5/12) level4
Puriko09	0.48 (12/25)	0.33 (4/12) level2	0.68 (17/25)	0.75 (9/12) level2
Spot	0.52 (12/23)	0.00 (0/12) level2	0.48 (10/21)	0.33 (4/12) level2
Tim	0.63 (15/24)	0.00 (0/12) level2	0.63 (15/24)	0.58 (7/12) level1
Yubisashi	1.00 (12/12)	0.58 (7/12) level1	1.00 (14/14)	0.58 (7/12) level1
GROUP	0.56 (120/220)	0.25 (27/108)	0.54 (114/217)	0.53 (57/108)

Data for subjects’ look proportion in the Tubes task were extracted from Subias et al. (2024). Fractions in parentheses indicate the number of trials in which subjects looked inside a tube divided by the total number of trials. The level numbers in the Impulsivity test columns indicate the heights at which the apparatus was set

**Fig. 2:**
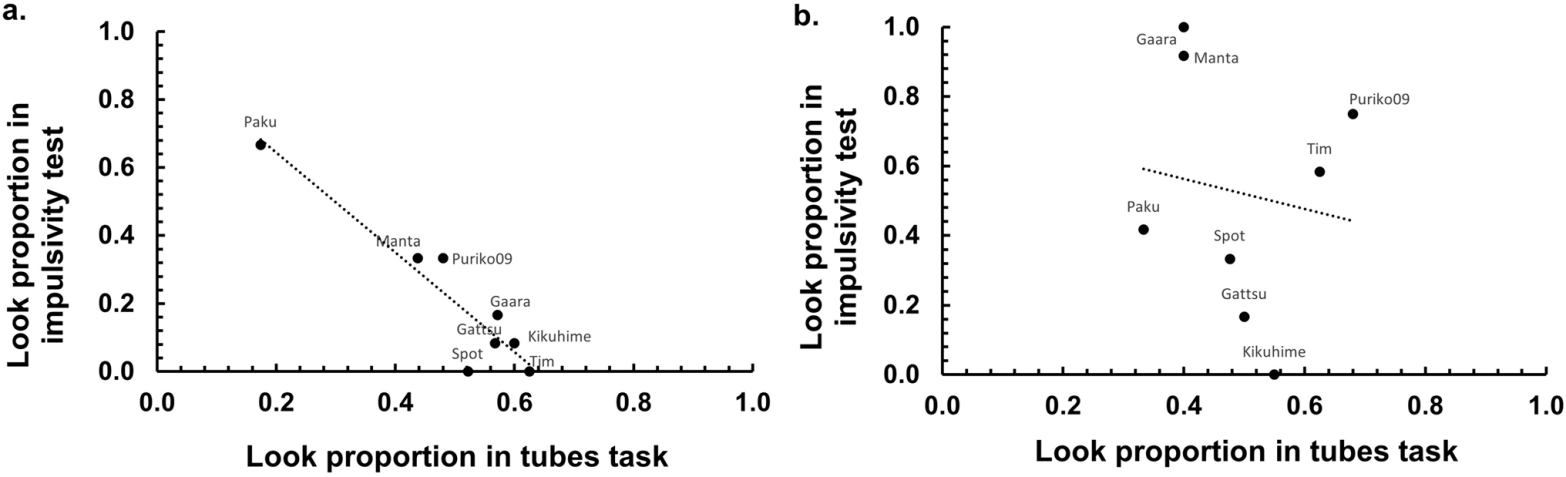
Correlation between the proportion at which monkeys looked inside the tubes in the tubes task when they knew the reward location (x-axis) and their look proportion in the impulsivity test (y-axis). Graph *a.* displays the result when using a low-quality reward, Graph *b.* when using a high-quality reward. Each dot represents an individual

Regarding the relationship between looking proportions in the impulsivity test and the tubes task, contrary to our a priori hypothesis, we found no positive correlation (Low-quality reward: Fig. 2a,* r*_*s*_ = − 0.76; High-quality reward: Fig. 2b, *r*_*s*_ = − 0.23), meaning that the monkeys who looked most frequently in the “known” condition of the tubes task did not show the highest look proportion in the single-tube reward situation (Table 1). Furthermore, the effect of reward quality on the correlation was not significant (*z* = − 1.477, *p* = 0.07).

However, we found a strong, significant negative correlation between looking in the impulsivity test and unnecessary looking in the tubes task when a low-quality reward was used, as an exploratory result (Fig. 2a: *r*_*s*_ = − 0.76, *N* = 8, *p* = 0.027). In the high-quality reward condition, the correlation was weak and non-significant (Fig. 2b: *r*_*s*_ = − 0.23, *N* = 8, *p* = 0.59).

## Discussion

We aimed to assess whether monkeys’ looking behavior in the tubes task could be explained by a desire to look at the reward. We found that Japanese macaques tended to look into a single tube they knew contained food they could not immediately reach. Looking occurred most frequently within the first five seconds following baiting, leading us to believe it was likely impulsive. Monkeys looked even more when a higher-quality reward was involved. Taken alone, this result suggests that the unnecessary looks observed in our monkeys in the tubes task (Subias et al. 2024) might come from an impulse to look at the reward. However, our a priori analyses did not demonstrate that the macaques’ looking proportion in the impulsivity test positively correlated with looking in the tubes task. Additionally, we did not find evidence to support a distinct correlation between looking in the impulsivity test and the likelihood of looking in the tubes task based on the quality of the reward. In opposition to our prediction, we found that the macaques displaying the highest rate of unnecessary looks in the four-tubes task displayed the lowest rate in the one-tube situation, thus revealing a strong negative correlation. The correlation became weak and non-significant when a highly preferred reward was involved; nevertheless, the absence of significant difference between the two correlations seems to indicate that reward quality did not have a strong impact on the correlation strength.

The seemingly contradictory nature of our findings prevents a definitive conclusion. A plausible interpretation could be that, in the tubes task, monkeys do not experience a strong impulse to look inside the tubes. Instead, reaching the tube might be the impulsive reaction animals must suppress to seek information. However, when selection is delayed, monkeys’ impulsivity may manifest as looking. This could explain monkeys’ looking behavior in the impulsivity test and why the looking proportion increases in the high-quality condition without positively correlating with looking proportions in the tubes task. Essentially impulsive subjects would tend to look in our impulsivity test but prefer to reach in the tubes task. Incorporating a condition where looking and reaching are explicitly in competition would offer clearer understanding.

It is also possible that subjects developed a habit of looking after completing the tubes task, although it is worth noting that a year separated the tubes task and impulsivity testing. Furthermore, even if they looked out of habit, this behavior would still be considered “impulsive” in this scenario.

While our impulsivity task does not directly assess inhibitory control since monkeys do not need to inhibit looking to perform a more appropriate behavior, a connection may exist between our subjects’ inhibitory capacities and their inclination to look in the impulsivity task. We hypothesize that individuals with weaker inhibitory control might exhibit more impulsive behavior. To validate this hypothesis, it would be essential to examine whether our impulsivity test correlates with measures of inhibition.

In the tubes task, macaques looked more frequently in the unknown condition than in the known condition. Regardless of the underlying mechanism (whether it be metacognitive processes, associative learning, response competition, or curiosity), subjects would be expected not to look in the known condition. However, our macaques looked in the known condition half the time. Subjects’ performance in this condition (99% success rate without looking; Subias et al. 2024), coupled with the observation that their first look was almost always directed toward the baited tube (94% of trials), makes it hard to believe they had forgotten the reward’s location. If looking behavior cannot be attributed to poor memory, then it might be that subjects sometimes fail to suppress the inappropriate looking response to make a direct selection. This could be a matter of inhibition. Although our impulsivity test does not directly measure inhibition, it revealed that our most impulsive subjects (in terms of looking inside a tube containing a reward) are not the ones who looked most frequently in the tubes task. Another possible explanation might be the “passport effect.” Currently, both possibilities remain valid, while other causes may still elude us.

At the very least, our experiment suggests that the factors prompting looking behavior in the one-tube situation differ from those in the tubes task. Despite the study's limitations, we find the results intriguing, and we believe that the question we attempted to address warrants further investigation. An important next step could involve measuring inhibition using a battery of validated tasks and exploring the relationship between inhibitory control and performance in metacognitive tests. In a recent paper, Kuhn (2022) emphasized the importance of inhibition in various domains requiring metacognition, such as theory of mind, understanding false belief, reasoning, and belief revision. More data investigating the relationship between metacognition and inhibition would greatly benefit our understanding of metacognition and help identify the type of inhibitory control involved in metacognitive processes.

## Supplementary Information

Below is the link to the electronic supplementary material.

Supplementary file1 Online Resource 1 Subjects included in the study. ~ means an approximate age, “un” stands for unknown and numbers under “Low” and “High” columns indicates whether subjects were tested at location 1 or 2 for low- and high-quality reward condition. The “First” column indicates which condition was tested first: low- or high-quality reward. (DOCX 17 KB)

## Data Availability

All data analyzed in this study are included in the published article and its supplementary information files.
