# Supplementary material for: Metacognition in Japanese macaques (Macaca fuscata): does impulsivity explain unnecessary looks in the tubes task?
Source: Anim Cogn. 2024 May 28;27(1):41. doi: 10.1007/s10071-024-01879-1 (PMC11133117; doi:10.1007/s10071-024-01879-1)
Supplement: Supplementary file 1 — Supplementary file1 Online Resource 1 Subjects included in the study. ~ means an approximate age, “un” stands for unknown and numbers under “Low” and “High” columns indicates whether subjects were tested at location 1 or 2 for low- and high-quality reward condition. The “First” column indicates which condition was tested first: low- or high-quality reward. (DOCX 17 KB) [file 10071_2024_1879_MOESM1_ESM.docx]

**SUPPLEMENTARY**

**Table 1** Subjects

| **Name** | **Age (years)** | **Sex** | **Rank** | **Low** | **High** | **First** |
| --- | --- | --- | --- | --- | --- | --- |
| Gaara | 15~19 | M | un | 2 | 2 | high |
| Gattsu | 18 | M | 6 | 1 | 1 | high |
| Kikuhime | ~20 | M | 7 | 1 | 2 | high |
| Manta | 16 | M | 15 | 1 | 2 | high |
| Paku | 15~19 | M | 12 | 1 | 2 | low |
| Puriko09 | 14 | F | 2 | 1 | 2 | low |
| Spot | 15~20 | M | un | 1 | 1 | low |
| Tim | 15~20 | M | un | 1 | 1 | high |
| Yubisashi | ~20 | M | un | 1 | 2 | low |

Subjects included in the study. ~ means an approximate age, “un” stands for unknown and numbers under “Low” and “High” columns indicates whether subjects were tested at location 1 or 2 for low- and high-quality reward condition. The “First” column indicates which condition was tested first: low- or high-quality reward.
